# The Development, Physicochemical Characterisation and *in Vitro* Drug Release Studies of Pectinate Gel Beads Containing Thai Mango Seed Kernel Extract

**DOI:** 10.3390/molecules18066504

**Published:** 2013-06-03

**Authors:** Saruth Nithitanakool, Pimolpan Pithayanukul, Sandrine Bourgeois, Hatem Fessi, Rapepol Bavovada

**Affiliations:** 1 Department of Pharmacy, Faculty of Pharmacy, Mahidol University, 447 Sri-Ayudthaya Road, Phayathai, Bangkok 10400, Thailand; E-Mail: saruth_pipe@yahoo.com; 2 University Lyon, University Claude-Bernard Lyon 1, LAGEP UMR CNRS 5007, F-69622, Villeurbanne, France; E-Mails: bourgeois@lagep.univ-lyon1.fr (S.B.); fessi@univ-lyon1.fr (H.F.); 3 ISPB-School of Pharmacy, University Lyon 1, F-69008, Lyon, France; 4 Department of Pharmaceutical Botany, Faculty of Pharmaceutical Sciences, Chulalongkorn University, Phayathai Road, Bangkok 10330, Thailand; E-Mail: rapepol1@hotmail.com

**Keywords:** colon-targeted delivery, mango seed kernel, calcium-pectinate gel beads, zinc-pectinate gel beads, ionotropic gelation, enteric-coated capsule

## Abstract

Pectinate gel beads containing Thai mango seed kernel extract (MSKE, cultivar ‘Fahlun’) were developed and characterised for the purpose of colon-targeted delivery. The MSKE-loaded pectinate beads were prepared using ionotropic gelation with varying pectin-to-MSKE ratios, MSKE concentrations, and concentrations of two cross-linkers (calcium chloride and zinc acetate). The formulated beads were spherical in shape and ranged in size between 1.13 mm and 1.88 mm. Zinc-pectinate (ZPG) beads containing high amounts of MSKE showed complete entrapment efficiency (EE) of MSKE (100%), while calcium-pectinate (CPG) beads demonstrated 70% EE. The *in vitro* release tests indicated that MSKE-loaded CPG beads were unstable in both simulated gastric medium (SGM) and simulated intestinal medium (SIM), while MSKE-loaded ZPG beads were stable in SIM but unable to prevent the release of MSKE in SGM. The protection of ZPG beads with gastro-resistant capsules (Eudragit^®^ L 100-55) resulted in stability in both SGM and SIM; they disintegrated immediately in simulated colonic medium containing pectinolytic enzymes. MSKE-loaded ZPG beads were stable at 4, 25 and 45 °C during the study period of four months. The present study revealed that ZPG beads in enteric-coated capsules might be a promising carrier for delivering MSKE to the colon.

## 1. Introduction

Phenolic compounds have attracted considerable attention for their beneficial effects on human health [1]. Mangos (*Mangifera indica* L.), which belong to the family *Anacardiaceae*, grow in tropical and subtropical regions, and their components are commonly used in folk medicine to produce a wide variety of remedies [2,3,4]. There are several reports available about the traditional uses of mango kernel in various parts of the World. In Fiji, fresh mango kernel is eaten for dysentery and asthma, while the juice is used in a nasal application for sinus trouble [3]. In India, dried seed powder is applied to the head to remove dandruff and the kernel starch is eaten as a famine food [5]. A hot water extract of the kernel is taken as an anthelmintic, aphrodisiac, laxative and tonic [2]. The pharmacological activities of mango seed kernel extract (MSKE) have also been studied [6]. Among the edible portions, MSKE has been reported to exhibit potent antioxidant activity and to have a relatively high phenolic content [7]. The ethanolic extract of Thai mango seed kernel cultivar ‘Fahlun’ and its major polyphenolic constituent, pentagalloylglucopyranose (PGG), have been shown to exhibit anti-methicillin-resistant *Staphylococcus aureus* and anti-tyrosinase properties, potent free radical scavenging, antioxidant and anti-inflammatory activities, as well as anti-hepatotoxicity activity against liver damage induced by carbon tetrachloride in rats [8,9,10]. PGG has been found to possess a broad spectrum of pharmacological activities. With its anti-oxidative and anti-inflammatory properties, it is a very promising novel drug candidate for the therapy and prevention of several diseases including cancer and diabetes [11].

The objectives of this study were to develop and characterise pectinate gel beads containing MSKE for the purpose of colon-targeted delivery. Colon drug delivery has the major advantage of utilising the colon for systemic therapy where the residence time is more than 24 h [12]. Moreover, the colon has a relatively mild environment with decreased fluid and motility, compared with the small intestine; this could provide advantages for the incorporation of multiple components in the formulation, such as absorption enhancers that must reach the epithelial absorptive layer in a high concentration and in close spatial proximity [13]. The dosage form of pectinate gel beads were selected and developed in this study because colonic microflora can degrade polysaccharides such as pectin; pectin is non-toxic and is not digested by gastric or intestinal enzymes, but pectin is almost completely degraded by pectinolytic enzymes produced by colonic microflora [14,15]. Pectin is composed of linear chains of α*-(1→4)-*d*-*galacturonic acid residues with carboxylic groups that are partially methoxylated. Some of the carboxylic groups may also be converted to carboxamide, producing amidated pectin. Pectin with low methoxylation [degree of methyl esterification (DE) < 50%], amidated or not, can form gel beads in a presence of divalent cations, such as Ca^2+^ and Zn^2+^ [16]. Through this mechanism, pectin was used to prepare cross-linkages between low-methoxy pectin with MSKE and different solutions of cross-linking agents (e.g., Ca^2+^ and Zn^2+^) to obtain Ca- or Zn-pectinate gel beads without the use of organic solvents and harsh ingredients as in the method described by Aydin and Akbuğa [16]. The resulting Ca-pectinate gel (CPG) beads and Zn-pectinate gel (ZPG) beads were characterised by morphological aspects and drug contents. Then, the beads were tested *in vitro* in dissolution conditions mimicking gastric to colon transit to check their efficacy to target the colon.

## 2. Results and Discussion

### 2.1. Preparation of MSKE-Loaded Pectinate Beads

In this study, MSKE was homogeneously dispersed in an aqueous solution of low methoxylated (LM) pectin and added dropwise to counter-ion solutions (e.g., Ca^2+^ and Zn^2+^). Intermolecular cross-links (known as the “egg-box” conformation) were instantaneously formed between the negatively charged carboxyl groups of the LM pectin molecules and the positively charged counter-ions. Consequently, the pectin droplets formed gelled spherical beads. This process is known as the ionotropic gelation method. Using this method, spherical beads were easily prepared, without the use of sophisticated instruments. 

In this study, we have prepared pectin beads using optimal concentrations of pectin and calcium chloride (CaCl_2_) at 6% (w/v) with a cross-linking time of 20 min to form stable and complete cross-linked pectin-Ca networks, as described by Bourgeois *et al*. [17]. The resulting MSKE-loaded pectinate gel beads were dark brown in colour.

### 2.2. Particle Size and Shape

The mean diameter and elongation ratios (ER) of the dried beads prepared with different formulation variables are shown in Table 1. Generally, the size of the beads was dependent on the diameter of the needle (0.8 mm inner diameter) used to prepare the beads, as wells as the drying method. The diameter of the needle and the drying method were constant throughout the experiment. Therefore, the resulting particle sizes of the dried beads prepared in this study were affected by other variables of the formulation.

For MSKE-loaded CPG beads, the mean diameter was increased from 1.13 ± 0.05 mm to 1.63 ± 0.10 mm as the pectin:MSKE ratio was increased from 1:1 to 1.75:1. An increase in pectin concentration generally leads to an increased mean bead diameter. When beads were prepared with a constant ratio of pectin:MSKE at 1:1 but with increases in both concentrations from 4:4% to 7:7% (w/w), the bead sizes (1.13 ± 0.05 mm–1.54 ± 0.10 mm) were in the same range of the beads prepared with an increasing ratio of pectin:MSKE. These results indicated that bead size was independent of MSKE concentration but dependent on pectin concentration.

It was found that an increase in the concentration of CaCl_2_ from 6% to 10% w/v did not significantly (*p* > 0.05) change the mean diameter of the CPG beads (1.54 ± 0.10 mm–1.70 ± 0.10 mm). This finding was because excess free Ca in the medium was eliminated from the beads.

ZPG beads were prepared with a constant ratio of pectin and MSKE (1:1), at 7% w/v each, but with increasing concentrations of zinc acetate (Zn(CH_3_COO)_2_); their size ranged between 1.86 ± 0.02 mm and 1.88 ± 0.01 mm. In this study, we found no significant differences (*p >* 0.05) in size ranges between ZPG beads and CPG beads (1.54 ± 0.10 mm–1.70 ± 0.10 mm) prepared with the same concentrations of pectin and MSKE (7% w/v).

**Table 1:** The effects of formulation parameters on particle size, shape and EE of MSKE-loaded pectinate beads.

Formulation parameters			Size (mm ± SD) *n* = 30	Shape (ER ± SD) *n* = 30	EE ^j^ (%)
Constants	Variables	Values			
MSKE (4%w/v) CaCl_2_ (6% w/v)	Pectin:MSKE (w/w)	1.00:1.00	1.13 ± 0.05 ^a^	1.10 ± 0.01	42.52 ± 1.78 ^k^
		1.25:1.00	1.36 ± 0.02 ^b^	1.15 ± 0.01	49.80 ± 2.16 ^l^
		1.50:1.00	1.50 ± 0.01 ^c^	1.13 ± 0.01	54.43 ± 0.43 ^m^
		1.75:1.00	1.63 ± 0.10 ^d^	1.13 ± 0.05	58.36 ± 0.38 ^n^
CaCl_2_ (6% w/v)	Pectin:MSKE (w/w)	4:4	1.13 ± 0.05 ^A^	1.10 ± 0.01	42.52 ± 1.78 ^K^
		5:5	1.40 ± 0.04 ^B^	1.12 ± 0.03	53.21 ± 2.84 ^L^
		6:6	1.43 ± 0.02 ^C^	1.15 ± 0.02	61.42 ± 3.01 ^M^
		7:7	1.54 ± 0.10 ^D^	1.11 ± 0.03	69.65 ± 0.71 ^N^
MSKE (7% w/v) Pectin (7%w/v)	CaCl_2_(%w/v)	6	1.54 ± 0.10 ^e^	1.11 ± 0.03	69.65 ± 0.71 ^r^
		7	1.60 ± 0.05 ^e^	1.09 ± 0.01	67.69 ± 1.44 ^r^
		8	1.62 ± 0.10 ^e^	1.08 ± 0.03	67.17 ± 2.43 ^r^
		9	1.63 ± 0.01 ^e^	1.09 ± 0.02	67.21 ± 0.37 ^r^
		10	1.70 ± 0.10 ^e^	1.07 ± 0.02	65.21 ± 1.96 ^r^
MSKE (7% w/v) Pectin (7% w/v)	Zn(CH_3_COO)_2_ (%w/v)	1	1.86 ± 0.02 ^E^	1.00 ± 0.01	100.22 ± 0.53 ^R^
		2	1.87 ± 0.01 ^E^	1.01 ± 0.01	100.12 ± 1.59 ^R^
		4	1.86 ± 0.05 ^E^	1.00 ± 0.00	97.64 ± 5.6 2 ^R^
		6	1.88 ± 0.01 ^E^	1.01 ± 0.01	104.48 ± 8.15 ^R^

^a–d^ Significant difference (*p* < 0.05) among formulations prepared with different ratios of pectin:MSKE; ^A–D^ Significant difference (*p* < 0.05) among formulations prepared with constant ratios of pectin:MSKE (1:1) but with increases in both concentrations; ^e^ No significant difference (*p* > 0.05) among formulations prepared with different concentrations of calcium chloride; ^E^ No significant difference (*p* > 0.05) among formulations prepared with different concentrations of Zn(CH_3_COO)_2_; ^j^ EE = (A_ent_ /A_i_) × 100%; where A_i_ = the initial amount of MSKE and A_ent_ = the amount of MSKE entrapped in the beads calculated as (B_rel_) × (1/B_cont_); where B_rel_ = amount of PGG released, B_cont_ = amount of PGG content in MSKE; ^k–n^ Significant difference (*p* < 0.05) among formulations prepared with different ratios of pectin:MSKE; ^K–N^ Significant difference among formulations prepared with different concentrations of pectin and MSKE while keeping their ratio constant at 1:1; ^r^ No significant difference (*p* > 0.05) among formulations prepared with different concentrations of CaCl_2_; ^R^ No significant difference (*p* > 0.05) among formulations prepared with different concentrations of Zn(CH_3_COO)_2_.

### 2.3. Scanning Electron Microscopy (SEM)

**Figure 1:**
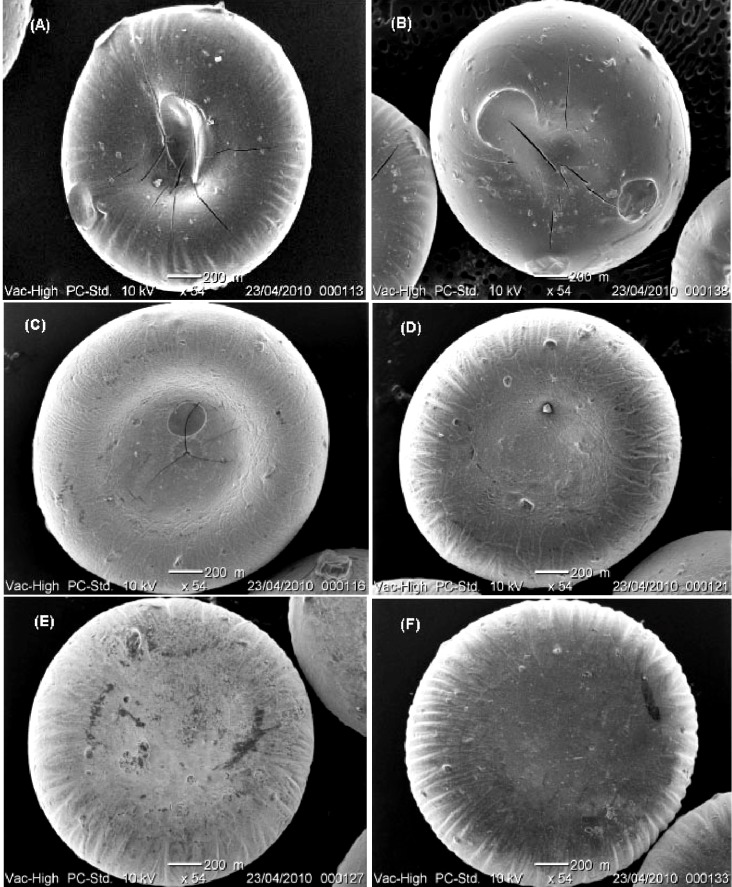
Scanning electron micrographs of MSKE-loaded pectinate beads (×54). (**A**) CPG beads prepared with 6% CaCl_2_; (**B**) CPG beads prepared with 10% CaCl_2_; (**C**) ZPG beads prepared with 1% Zn(CH_3_COO)_2_; (**D**) ZPG beads prepared with 2% Zn(CH_3_COO)_2_; (**E**) ZPG beads prepared with 4% Zn(CH_3_COO)_2_; and (**F**) ZPG beads prepared with 6% Zn(CH_3_COO)_2_.

**Figure 2:**
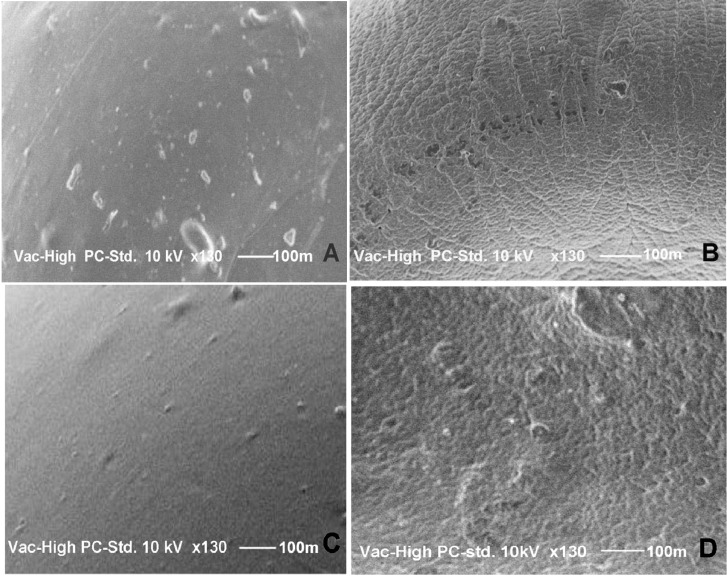
SEM of the pectinate bead surfaces (130 × magnification). (**A**) MSKE-free zinc-pectinate bead; (**B**) MSKE-loaded zinc-pectinate bead; (**C**) MSKE-free calcium-pectinate bead; (**D**) MSKE-loaded calcium-pectinate bead.

The SEM photographs (Figure 1) indicate that the ZPG beads were more spherical in shape than the CPG beads. These results correspond to the ER values shown in Table 1. This could be due to the flattening and collapsing of CPG beads during the drying process. The surfaces of the beads differed according to the counter-ion used. Figure 2 shows SEM images of the MSKE-free beads with smooth surface topography for both CPG (Figure 2C) and ZPG (Figure 2A]. Conversely, the MSKE-loaded beads were characterised by rough surfaces (Figure 2B,D), which might be due to the presence of MSKE embedded in the bead matrix.

### 2.4. Entrapment Efficiency (EE)

Table 1 shows the effects of formulation parameters on the EE of MSKE of Ca- and Zn-pectinate beads. For MSKE-loaded CPG beads, the percent EE increased from 43% to 58% as the ratio of pectin:MSKE was increased from 1:1 to 1.75:1. The percent EE also increased from 43% to 70% with the increase in pectin and MSKE concentrations from 4% to 7% (w/v) while keeping their ratio constant at 1:1. Increasing the concentration of pectin generally leads to a higher EE due to the increased polymer-to-drug ratio. Furthermore, a more compact gel network structure can be formed at higher pectin concentrations, and therefore, the diffusional loss of MSKE during bead formation could be reduced. In this study, the variation of CaCl_2_ concentrations from 6% to 10% w/v did not significantly (*p* > 0.05) change the MSKE EE (65%–70%) of the CPG beads. 

Based on the above findings, the CPG beads demonstrated the greatest EE of MSKE at 70% with concentrations of pectin and MSKE both at 7% w/v. The viscosity of the pectin and MSKE mixture at this concentration did not cause the needle to clog during the preparation of pectin beads. These appropriate conditions were therefore chosen for the preparation of the MSKE-loaded ZPG beads. 

A significant improvement in the EE was observed when Zn(CH_3_COO)_2_ was used to prepare MSKE-loaded pectinate beads (Table 1). CPG beads demonstrated EE of MSKE at 43%–70%, while ZPG beads showed complete entrapment of MSKE (100%) at all of the concentrations used (1, 2, 4 and 6% w/v). Better encapsulation efficiency of ZPG beads was probably because the rate of Zn^2+^ in producing a pectinate cross-linking was faster than Ca^2+^. This led to the reduction of the diffusional loss of MSKE during the bead formation, resulting in very high percentages of MSKE encapsulation of ZPG beads.

### 2.5. *In Vitro* Release Studies of MSKE-Loaded Pectinate Beads

The *in vitro* release studies in simulated gastric medium (SGM) and simulated intestinal medium (SIM) were carried out to determine the feasibility of different beads (CPG or ZPG) to protect against the release of MSKE in gastric and intestinal media from the upper part of the gastrointestinal tract and allow release specifically in the colon target. 

**Figure 3:**
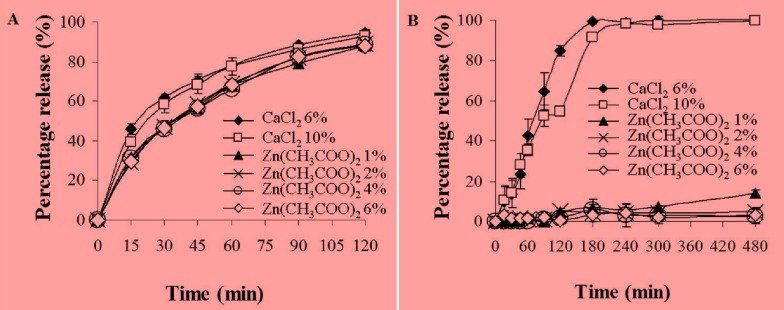
Effects of the type of cross-linker (CaCl_2_ or Zn(CH_3_COO)_2_) and cross-linker concentration on the release of MSKE from CPG and ZPG beads determined after incubation in SGM for 2 h (**A**) and SIM for 8 h (**B**) at 37 °C. Data are presented as the mean ± SD of three determinations.

Figure 3A shows the percentage release of MSKE from the pectinate beads determined after 2 h of incubation in SGM at 37 °C. It was found that the percentage release of MSKE from the CPG beads was not significantly different than from the ZPG beads (*p* > 0.05) at 2 h of incubation. The majority of the entrapped MSKE (88%–95%) was already released from the CPG and ZPG beads in SGM after 2 h of incubation. Moreover, it was observed that the release of MSKE from these beads into SGM was not affected by the concentrations of the cross-linking agents (CaCl_2_ and Zn(CH_3_COO)_2_). These results may be attributed to a drastic and sudden swelling of the dried pectinate beads, which occurred after they were exposed to SGM. However, the beads apparently kept their structure intact due to the decreased solubility of pectinic acid, a product from the ion exchange in the SGM. 

When the pectinate beads were incubated in SIM for 8 h, the percentage release of MSKE from the CPG beads was significantly different than from the ZPG beads (*p* < 0.05), as shown in Figure 3B. The entrapped MSKE was completely released (100%) from the CPG beads into SIM after 4 h of incubation because of significant degradation of the bead structure. Conversely, the ZPG beads did not disintegrate and only a small amount of MSKE (5%–15%) was released after 8 h of incubation. These results are consistent with those of El-Gibaly [18], Atyabi *et al.* [19] and Chambin *et al.* [20] who reported differences in the degree of cross-linking with the two pectinate gel types and that the CPG beads displayed more drastic swelling-erosion in the intestinal medium than the ZPG beads. Calcium ions could possibly form loose linkages with carboxyl groups in the pectin chains, allowing the penetration of SIM into the Ca-pectinate network. Then, ion exchanges between Ca^2+^ and Na^+^ or K^+^ ions could occur, which might result in a swelled bead structure [21]. In addition to that, the capture of the Ca^2+^ ions by phosphate ions present in the SIM changed the equilibrium towards this medium which further accelerated the disintegration of the pectinate bead structure [22]. Conversely, the use of zinc ions resulted in extensive cross-linking with LM pectins, with a higher binding affinity and higher selectivity than calcium ions [23]. This resulted in a reduction in both the extent of rehydration and molecular porosity of the zinc-pectinate network [18]. Thus, ZPG beads remained intact and did not disintegrate after 8 h of incubation in SIM. Therefore, the ZPG beads were more appropriate than the CPG beads as a carrier for colonic delivery of MSKE because the ZPG beads were more stable in the upper part of gastrointestinal tract than the CPG beads.

Table 2 shows that after 13 h of incubation in simulated colonic medium (SCM) without pectinolytic enzymes, a complete (100%) release of the entrapped MSKE was obtained from the ZPG beads prepared with 1% Zn(CH_3_COO)_2_ while only 3%–4% of MSKE was released from the ZPG beads prepared with 2, 4, and 6% Zn(CH_3_COO)_2_. This result implies that the ZPG beads prepared with 1% Zn(CH_3_COO)_2_ were inappropriate for colon-specific delivery of MSKE. In the presence of pectinolytic enzymes, the percentage release of MSKE occurred to a greater extent (25%) from the ZPG beads prepared with 2% Zn(CH_3_COO)_2_ compared to the 3% release of MSKE from the ZPG beads prepared with 4 and 6% Zn(CH_3_COO)_2_. These results indicated that the release of MSKE from the ZPG beads into SCM was dependent on the concentrations of pectinolytic enzymes in the medium and of Zn(CH_3_COO)_2_ used to prepare the beads. 

**Table 2:** The percentage release of MSKE from ZPG beads prepared with different concentrations of Zn(CH_3_COO)_2_ solution in SCM with the absence or presence of pectinolytic enzymes (650 U/mL) after incubation for 13 h at 37 °C. (Data represent the mean ± SD, *n* = 3).

	Percentage release of MSKE with different % Zn(CH_3_COO)_2_			
	1%	2%	4%	6%
SCM without enzymes	101.14 ± 2.20	4.00 ± 0.56	3.04 ± 0.65	3.16 ± 0.21
SCM with enzymes; 650 U/mL	NT	25.55 ± 0.78	3.15 ± 1.19	3.13 ± 0.47

NT = Not Tested.

**Figure 4:**
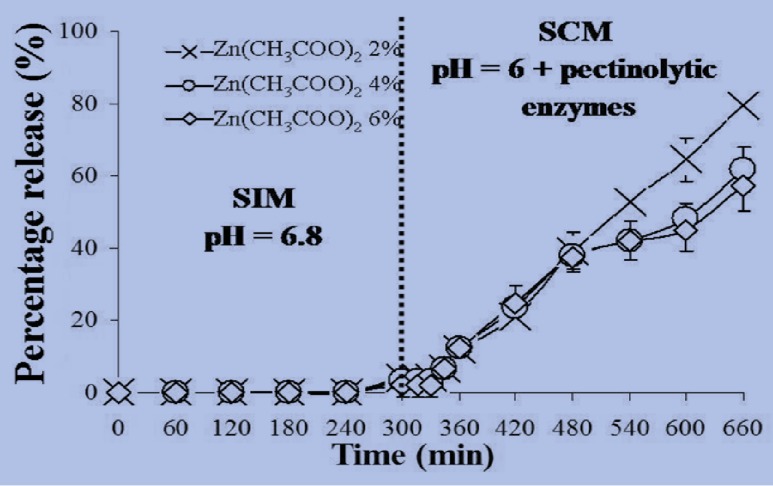
The release of MSKE from ZPG beads prepared with different concentrations of Zn(CH_3_COO)_2_ (2, 4 and 6% w/v) determined after a 5 h-pre-incubation in SIM and a 6 h-incubation in SCM containing pectinolytic enzymes (650 U/mL). Data are presented as the mean ± SD of three determinations.

It is well known that the oral dosage formulations must pass through the small intestine before reaching the colon. To mimic the GI tract conditions, the release of MSKE from MSKE-loaded ZPG beads was investigated by pre-incubation in SIM for 5 h before transfer to SCM containing pectinolytic enzymes (650 U/mL) for the next 6 h of incubation at 37 °C. Figure 4 illustrates that small amounts of MSKE were released (2%–4%) from the ZPG beads prepared with 2, 4 and 6% Zn(CH_3_COO)_2_ after 5 h of incubation in SIM. Nevertheless, the pre-incubation of these beads in SIM contributed to the loosening of the gel-network structure in SCM where 57%–80% of the entrapped MSKE was released after 6 h of incubation. Because beads were disintegrated in SCM by the activity of pectinolytic enzymes, the pre-incubation of the beads in SIM would lead to an ion exchange between Zn^2+^ and Na^+^ or K^+^ from SIM. As a result, the egg-box structure of the beads would loosen and form soluble pectin regions, which are more permeable to pectinolytic enzyme access, resulting in the degradation of the pectinate network.

**Figure 5:**
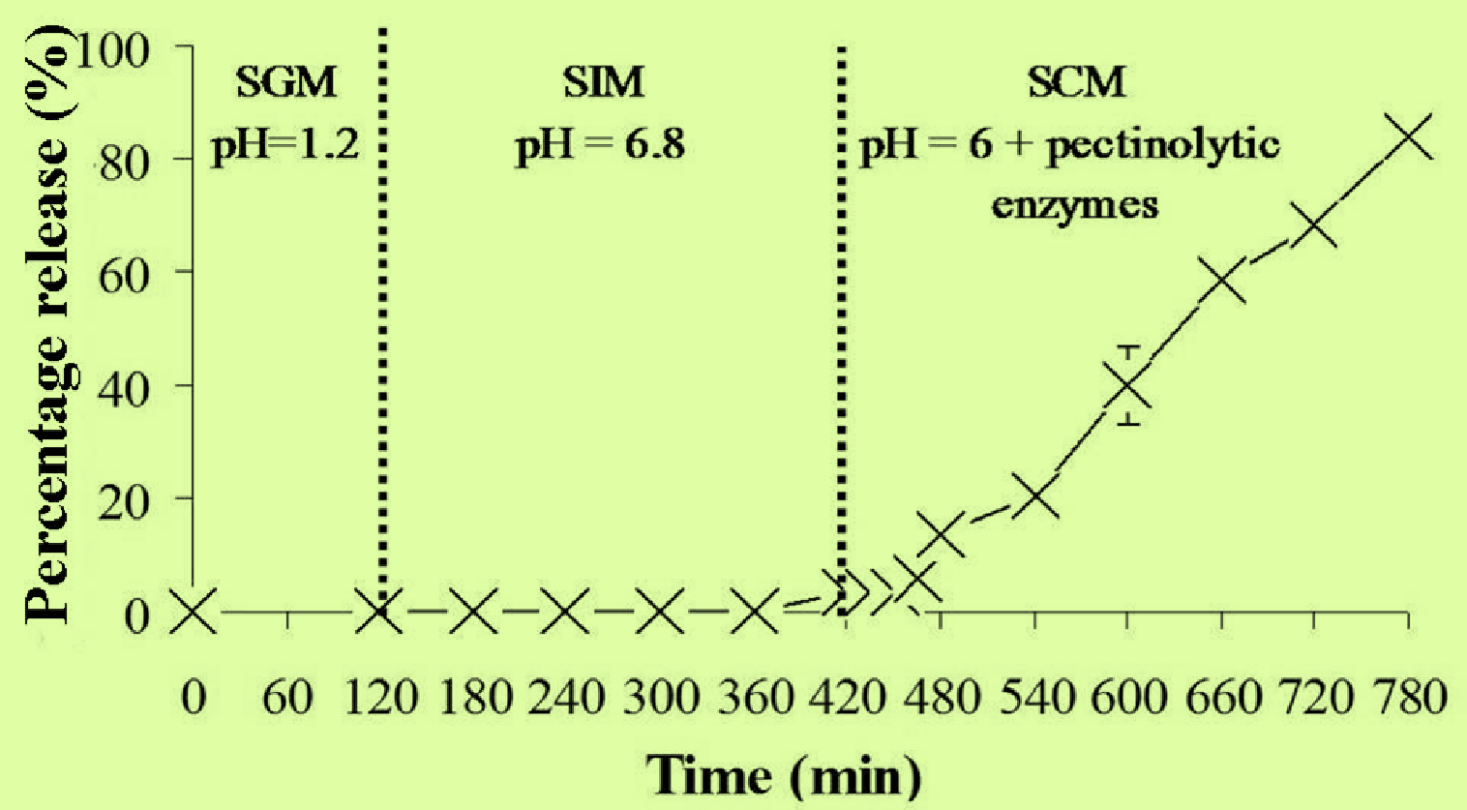
Release profile of MSKE from ZPG 2% beads in enteric-coated capsules determined under conditions mimicking the gastro-intestinal tract (pH 1.2 then pH 6.8) to the colon (pH 6 with pectinolytic enzymes). Data are presented as the mean ± SD of three determinations.

It has been reported that the transit time in the portion of the colon favourable to absorption was about 19 h [24]. The results depicted in Figure 4 clearly show that all the entrapped MSKE would be completely released from the ZPG beads in the colon within this 19 h period. Therefore, it seems reasonable to propose that the ZPG beads are feasible as a carrier for specific delivery of MSKE to the colon. It was found that the gastro-resistant capsules, coated with Eudragit^®^ L 100-55 and containing the ZPG beads prepared with 2% Zn(CH_3_COO)_2_ were resistant to SGM without disintegration of the beads or the release of MSKE after 2 h of incubation (Figure 5). However, the coated capsules did disintegrate in SIM, and the beads were released after an incubation of only 12 ± 1.63 min. The released beads were found to be stable under SIM conditions and only small amounts of MSKE (3%) were released after 5 h of incubation. In SCM containing pectinolytic enzymes, the disintegration of the ZPG beads did occur and up to 84% of the entrapped MSKE was released after 6 h of incubation. This could be due to significant degradation of the bead structure by the activity of pectinolytic enzymes.

### 2.6. Stability Study

The ZPG beads could maintain the initial EE of MSKE (99%–100% EE) after storage for 4 months at the three different temperatures studied (Table 3). This finding indicated that the MSKE was stable inside the ZPG beads. This result suggests that the inclusion of MSKE within the ZPG beads did not affect bead stability and demonstrates the long-term stability of MSKE in these beads.

**Table 3:** EE and stability evaluation of MSKE in ZPG beads prepared with different percents of Zn(CH_3_COO)_2_ at 4, 25 and 45 °C after storage for four months. (Data represent the mean ± SD, *n* = 3).

Temperature	% EE of MSKE with different % Zn(CH_3_COO)_2_		
	2%	4%	6%
4 °C	98.78 ± 1.89	99.55 ± 1.73	98.57 ± 0.79
25 °C	99.33 ± 0.33	99.65 ± 0.88	100.85 ± 1.64
45 °C	99.46 ± 1.19	99.95 ± 2.36	99.53 ± 0.98

## 3. Experimental

### 3.1. Chemicals

Low methoxylated (LM) non-amidated pectin (Unipectine^TM^ OF400, DE from 27% to 32%) was obtained from Cargill (Redon, France). Calcium chloride dihydrate and ethylenediaminetetraacetic acid (EDTA) were obtained from Acros Organics (Geel, Belgium). Zinc acetate dihydrate, pepsin, pancreatin from porcine pancreas, pectinolytic enzymes (Pectinex^®^ Ultra SPL; 10,454 U/mL), which is a mixture of pectinases from *Aspergillus aculeatus*, and other simulated fluids constituents (NaCl, HCl, KH_2_PO_4_, NaOH, and Tween^®^ 80) were purchased from Sigma-Aldrich (Saint-Quentin-Fallavier, France). Eudragit^®^ L 100-55 (methacrylic acid-ethyl acrylate copolymer) was obtained from Evonik (Darmstadt, Germany). Pentagalloylglucopyranose (PGG; >95%) was obtained from Endotherm GmbH (Saarbrücken, Germany). Purified water (18.2 M Ω cm at 25 °C) was obtained from a Millipore Synergy^®^ ultrapure water system (Molsheim, France). High-performance liquid chromatography (HPLC) grade solvents were obtained from Fisher Scientific (Leicestershire, UK). Simulated digestive media were prepared according to the USP28-NF23 (2005) as follows: 

**Simulated gastric medium (SGM)** was prepared by dissolving 2 g of NaCl and 3.2 g of purified pepsin in 7 mL of HCl and sufficient water to make 1,000 mL. The final pH was 1.2.

**Simulated intestinal medium (SIM)** was prepared by dissolving 6.8 g of KH_2_PO_4_, 0.4% (w/v) Tween^®^ 80, 77 mL of 0.2 N NaOH and 10 g of pancreatin in sufficient water to make 1,000 mL. The pH was adjusted to pH 6.8 with 0.2 N NaOH.

**Simulated colonic medium (SCM,** containing pectinolytic enzyme 650 U/mL) was prepared by dissolving 6.8 g of KH_2_PO_4_, 0.4% (w/v) Tween^®^ 80, 77 mL of 0.2 N NaOH and 62.18 mL of pectinolytic enzymes (10454 U/mL) in sufficient water to make 1,000 mL. The pH was adjusted to 6 with concentrated HCl solution. The SCM without pectinolytic enzymes was prepared in the same manner.

### 3.2. Preparation of MSKE

Fully grown, unripened Thai mango fruits (*Mangifera indica* L. cultivar ‘Fahlun’, *Anacardiaceae*) were purchased from a local market. The seed kernels were removed and extracted following the method described by Nithitanakool *et al.* [8]. Briefly, the kernels were homogenised in hot ethanol (80 °C) and defatted with hexane. After the solvents were evaporated, the remaining aqueous residue was freeze-dried and stored in a vacuum desiccator at 4 °C until use.

### 3.3. Analysis of PGG Content in MSKE

**Figure 6:**
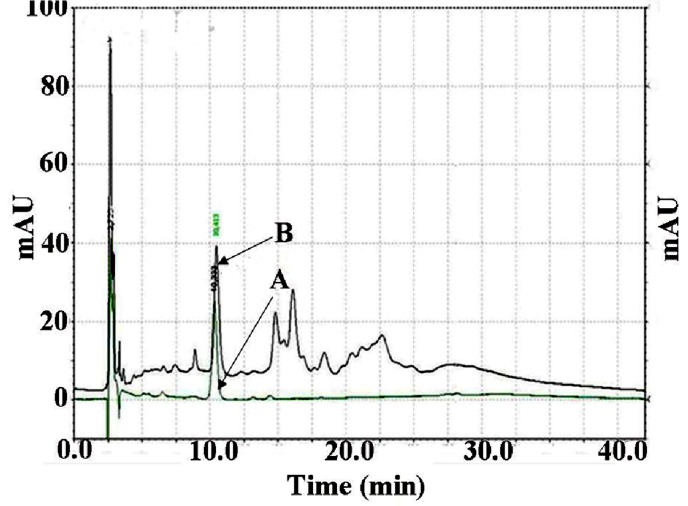
HPLC chromatograms of the reference standard pentagalloylglucopyranose (PGG), retention time (RT) = 10.3 min (A); and PGG present in mango seed kernel extract, RT = 10.4 (B). The stationary phase was a Gemini-NX C-18 column (250 × 4.6 mm, 5 μm particle size) with a guard column. The mobile phase consisted of 0.48% phosphoric acid in a mixture of 80/20 (v/v) water/acetonitrile (solvent A) and 0.48% phosphoric acid in a mixture of 70/30 (v/v) water/acetonitrile (solvent B). Total running time was 42 min, and the gradient program was set as follows: 100% A (5 min), 100% A to 38.4% A (22 min), 38.4% A to 100% A (15 min). The flow rate was 1 mL/min at room temperature (25 °C). The injection volume was 20 μL. The UV detector was set at 280 nm.

The quantitative determination of PGG content in MSKE was carried out by using HPLC (Spectra System; Thermo Separation Products, Yokohama, Japan) equipped with a P1000XR pump, a SCM1000 vacuum degasser, an AS3000 autosampler and a UV6000 LP detector. The data were recorded and analysed with Chromquest^®^ PC software (Thermosystems Inc., Lombard, IL, USA) with the Spectra System SN4000 unit. The Gemini-NX column (C18; 250 × 4.6 mm i.d., particle size 5 µm; Phenomenex Inc., Torrance, CA, USA) with a guard column was used as a stationary phase. The elution gradient for the HPLC analysis consisted of two solvent compositions: 0.48% phosphoric acid in a mixture of 80/20 (v/v) water/acetonitrile (solvent A) and 0.48% phosphoric acid in a mixture of 70/30 (v/v) water/acetonitrile (solvent B). Total running time was 42 min, and the gradient program was set as follows: 100% A (5 min), 100% A to 38.4% A (22 min), 38.4% A to 100% A (15 min). The flow rate was 1 mL/min at room temperature (25 °C). The samples (MSKE and the reference standard, PGG) were dissolved in dimethylsulfoxide (DMSO), and the sample injection volume was 20 µL. UV detection was carried out by monitoring the absorbance signal at 280 nm. Identification of PGG in MSKE was based on the comparison of the retention time and UV spectrum of the unknown peaks with the reference standard, PGG (Figure 6). The amount of PGG present in MSKE (100.03 ± 1.64 mg/g dry weight) was calculated using a calibration curve of the reference standard PGG with a concentration range of 0.3125–20 µg/mL. The linear equation of the calibration curve was y = 113296 x + 875.21. The linear correlation coefficient was 0.9998 for the line. All experiments were performed in triplicate.

### 3.4. Preparation of MSKE-Loaded Pectinate Gel Beads

The MSKE-loaded pectinate beads were prepared by ionotropic gelation using CaCl_2_ dihydrate and Zn(CH_3_COO)_2_ dihydrate as cross-linking agents [16,17,18]. Briefly, pectin was dissolved in distilled water (10 mL). MSKE was homogeneously dispersed in the aqueous solution of pectin with the aid of a magnetic stirrer. Using a peristaltic pump, the pectin-MSKE mixture was then dropped slowly (0.64 mL/min) through the blunt end of a 21-G needle (0.8 mm inner diameter) from a 10 cm falling distance into a gently agitated cross-linking solution (40 mL) at room temperature (25 °C). Upon contact with cross-linking solution, pectin droplets instantly formed pectinate gel beads. These beads were allowed to stand in a mild agitated cross-linking solution for 20 min for further cross-linking of the beads. After 20 min, the beads were separated by filtration and subsequently washed with distilled water to eliminate excess free Ca^2+^ and Zn^2+^ retained in the beads. Then, these beads were dried at 37 °C for 5 h in an oven. The beads were prepared by differing the formulation variables (pectin-to-MSKE ratio, MSKE concentration, CaCl_2_ concentration and Zn(CH_3_COO)_2_ concentration) as follows:

(i) Varying the pectin-to-MSKE ratios (1:1, 1.25:1, 1.5:1 and 1.75:1; w/w), while keeping the concentrations of MSKE and CaCl_2_ constant at 4 and 6% w/v, respectively.

(ii) Varying the MSKE concentration (4, 5, 6 and 7% w/v), while keeping the pectin-to- MSKE ratio (1:1 w/w) and CaCl_2_ concentration (6% w/v) constant.

(iii) Varying the CaCl_2_ concentration (6, 7, 8, 9 and 10% w/v), while keeping the pectin and MSKE concentrations constant at 7% w/v

(iv) Varying the Zn(CH_3_COO)_2_ concentration (1, 2, 4 and 6% w/v), while keeping the pectin and MSKE concentrations constant at 7% w/v.

All batches were prepared in triplicate.

### 3.5. Characterization of Pectin Beads

The effects of the formulation parameters on the bead characteristics were investigated.

#### 3.5.1. Particle Size and Shape

The size and shape of the dry beads were measured using an optical microscope (Motic^®^ BA 300, Wetzlar, Germany). In each batch, thirty beads were randomly selected for the study. After images of the beads were captured through a digital camera (Moticam 2300) connected with a microscope, the length and breadth of each bead were measured using the pre-calibrated image analysis program (Motic Image Plus, version 2.0). The size of each bead was calculated from the average of these two dimensions [25]. The average size of the beads from each batch was expressed as the mean diameter (mm) ± standard deviation (SD).

The shape of the beads was represented by the ER, which was the quotient of length to breadth of the beads [25]. An ER value of unity (ER = 1) represents a perfect spherical shape, while ER values between 1 and 1.15 indicate more deviation from a spherical shape. A non-spherical shape is represented by ER values > 1.15.

#### 3.5.2. SEM

Morphological examination of the pectinate gel beads was conducted by SEM at an excitation voltage of 10 kV. Dry beads were deposited on carbon conductive double-sided tape (Agar Scientific, France), and images were recorded at magnifications of 54× and 130×.

#### 3.5.3. Determination of EE

Ten beads (Ca- or Zn-pectinate network) were dissolved in 10 mL of 1% w/v EDTA aqueous solution with continuous stirring by a magnetic stirrer. DMSO (3 mL) was added to the system to ensure solubilisation of MSKE and the mixture was mixed well using a magnetic stirrer. The mixture was centrifuged at 10,000 rpm to remove pectin. An aliquot (1 mL) of the supernatant was diluted with DMSO (5 mL). PGG content in the supernatant was determined by HPLC. The actual amount of PGG present in the beads was calculated from the calibration curve constructed by plotting various concentrations of the reference standard PGG in DMSO (0.5–32 µg/mL) versus the peak areas. The linear correlation coefficient was 0.9999 for the line. The actual amount of MSKE entrapped in the beads (A_ent_) was calculated from the content analysis of PGG in MSKE (B_cont_):

A_ent_ = B_rel_ × (1/B_cont_)

where B_rel_ = amount of PGG released from the beads into the supernatant, and B_cont_ = amount of PGG content in MSKE (100 mg of PGG per g of MSKE).

Beads prepared without MSKE were used as blank controls. All experiments were performed in triplicate. The EE of pectinate beads for MSKE was calculated according to the following equation:

EE (%) = (A_ent_/A_i_) × 100%

where A_i_ was the initial amount of MSKE added into the pectin aqueous solution.

#### 3.5.4. *In Vitro* Release Studies of MSKE-loaded Pectinate Beads

The *in vitro* release tests were performed in water baths at 37 °C with SGM, SIM, and SCM. Briefly, MSKE loaded-pectinate beads, equivalent to ~8 mg of MSKE, were introduced into screw cap glass bottles (60 mL) containing 25 mL of releasing medium (preheated at 37 °C). For each releasing medium, separate bottles were employed for withdrawing samples at each specified time interval during the study periods.

In the first set of experiments, beads were placed in SGM bottles for 2 h in a water bath with continuous tangential stirring using a magnetic stirrer. Samples (2 mL) were withdrawn at each specified time interval (0, 15, 30, 45, 60, 90 and 120 min) and centrifuged at 10,000 rpm for 5 min before analysing the PGG content by HPLC. Similarly, for the second and the third sets of experiments, the PGG-release tests of beads in SIM and SCM bottles were carried out during incubation periods of 8 h and 13 h, respectively. For the fourth set of experiments, beads were first incubated for 5 h in SIM bottles before being transferred into SCM bottles containing pectinolytic enzymes (650 U/mL) for the next 6 h of incubation. The last set of experiments was carried out with gastro-resistant coated capsules (size 0) containing MSKE-loaded ZPG beads prepared with 2% Zn(CH_3_COO)_2_ (equivalent to ~8 mg of MSKE). The coating of the capsules was performed by dipping and drying capsules 5 times in an acetone solution containing 10% (w/w) Eudragit^®^ L 100-55. The coated capsules containing pectinate beads were first incubated for 2 h in SGM bottles before being transferred into SIM bottles and were then incubated until the disintegration of coated capsules was observed. The beads were further incubated in SIM for 5 h before being transferred into SCM containing pectinolytic enzymes for the next 6 h of incubation. At each incubation period, samples were taken at specified time intervals for PGG content analysis.

The PGG content in each releasing medium was calculated from the calibration curves constructed by plotting various concentrations of the reference standard PGG in SGM (4–64 µg/mL), SIM (4–96 µg/mL) and SCM containing pectinolytic enzymes (8–96 µg/mL) versus the peak areas. The linear correlation coefficients were 0.9996, 0.9996 and 0.9999 for the lines in SGM, SIM and SCM, respectively. The percentage release of MSKE was calculated by the following equation:

Percentage release of MSKE (%) = (A_ent_/A_i_) × 100

where A_rel_ was the amount of MSKE released from beads into the simulated medium and:

A_rel_ = (B_rel_) × (1/B_cont_)

where B_rel_ = amount of PGG released from beads into the simulated medium, B_cont_ = amount of PGG content in MSKE (100 mg PGG per g of MSKE). A_i_ was the initial amount of MSKE entrapped in the beads (~8 mg). All of the above experiments were performed in triplicate.

### 3.6. Stability Study

The MSKE-loaded ZPG beads prepared with 2%, 4% and 6% of Zn(CH_3_COO)_2_ were transferred into 5 mL glass vials with rubber stoppers (10 beads per vial) and were stored at three different controlled temperatures: cold (4 °C), room temperature (25 °C), and accelerated temperature (45 °C) for 4 months (120 days). On day 121, ten beads from the vials at the different temperatures were used to measure MSKE EE by using the same procedure described in the EE study. Stability was assessed by comparing the initial EE with that obtained after storage for 4 months at each temperature. All samples were performed in triplicate. 

### 3.7. Statistical Analysis

All data were expressed as the mean value ± SD. Significant differences (*p* < 0.05) among the corresponding mean values were determined by using one-way analysis of variance (ANOVA) followed by Tukey’s pairwise comparison test. SPSS 14.0 for Windows was used for statistical analysis.

## 4. Conclusions

For formulations containing high amounts of MSKE at pectin:MSKE = 1:1, ZPG beads showed higher entrapment efficiency of MSKE than CPG beads. The *in vitro* release studies revealed that although ZPG beads were unable to prevent the release of MSKE into SGM, the MSKE-loaded ZPG beads were more stable in the upper part of GI tract than the MSKE-loaded CPG beads. The protection of ZPG beads in enteric-coated capsules (Eudragit^®^ L 100-55) resulted in stability against both SGM and SIM but disintegrated immediately in SCM containing pectinolytic enzymes. MSKE-loaded ZPG beads were stable at all temperatures (4, 25 and 45 °C) during the study period. The results obtained implied that ZPG beads may be a promising carrier for colon-targeted delivery of MSKE.
